# Bioinformatic Selection of Mannose-Specific Lectins from *Allium genus* as SARS-CoV-2 Inhibitors Analysing Protein–Protein Interaction

**DOI:** 10.3390/life15020162

**Published:** 2025-01-23

**Authors:** Stefan Isaković, Milan Senćanski, Vladimir Perović, Kristina Stevanović, Ivana Prodić

**Affiliations:** 1 Independent Researcher, 11222 Belgrade, Serbia; isakovic991@gmail.com; 2 Laboratory of Bioinformatics and Computational Chemistry, Institute of Nuclear Sciences Vinca, National Institute of the Republic of Serbia, University of Belgrade, 11001 Belgrade, Serbia; vladaper@vin.bg.ac.rs (V.P.); kristina.stevanovic@vin.bg.ac.rs (K.S.); 3 Department of Computational Biochemistry and Drug Design, National Institute of Chemistry, 1000 Ljubljana, Slovenia; 4 Institute of Virology, Vaccines and Sera “Torlak”, Vojvode Stepe 458, 11042 Belgrade, Serbia

**Keywords:** bioinformatics, informational spectrum method, lectins, spike protein, SARS-CoV-2

## Abstract

Mannose-specific lectins are carbohydrate-binding proteins known for their antiviral potential. This study uses a bioinformatic approach to investigate the possibility of lectins from *Allium sativum* (garlic) and *Allium ursinum* (wild garlic) as inhibitors of SARS-CoV-2 entry. The information spectrum method (ISM) identified key interaction frequencies between the SARS-CoV-2 spike protein and these lectins, explicitly targeting the receptor-binding domain (RBD) and glycosylated asparagine residues, including N234. Lectins from *Allium* species showed a high affinity for oligomannose-type glycans on the spike protein, potentially blocking virus entry by preventing the spike-ACE2 receptor interaction. We propose that Allium lectins are promising candidates for further experimental validation as SARS-CoV-2 inhibitors, offering potential therapeutic applications in managing viral infections.

## 1. Introduction

Lectins are a group of carbohydrate-binding proteins, highly appreciated for their ability to interact with specific sugar moieties on cell surfaces, playing crucial roles in various biological processes such as cell–cell interactions, immune response, and pathogen recognition [1]. The surface of cells and intracellular structures contains specific glycoconjugates that lectins recognise and bind to. Their potential use as target molecules for practical applications in food, agriculture, health, and pharmaceutical research is worth considering [2]. Plants, algae, fungi, and cyanobacteria all contain various forms of lectins, which have been demonstrated to have carbohydrate–binding properties [3]. Mannose-specific lectins have attracted considerable attention from the scientific community due to their antiviral potential, as they interact with viral envelope glycoproteins, with the primary outcome of such interaction as the inhibition of viral replication [4]. Several plant lectins (Cyanovirin, NICTABA) have demonstrated inhibitory effects against influenza A and B viruses by binding to viral hemagglutinin glycoproteins, thereby preventing viral entry and replication [4]. The antiviral properties of plant lectins were first identified when D-mannose-specific lectins were found to inhibit HIV binding in vitro [5].

Also, the review papers highlight the ability of lectins, especially mannose-specific lectins, to bind to glycans on the SARS-CoV-2 protein and inhibit viral entry. The review explores the antiviral mechanisms of lectins and their role in modulating glycosylation-dependent processes [1,5]. Grosche et al. [6] investigated the antiviral efficacy of ConBR and DVL, mannose-binding lectins from *Canavalia brasiliensis* and *Dioclea violacea*, against multiple SARS-CoV-2 variants (Wuhan-Hu-1, Gamma, and Omicron). Using a combination of in vitro experiments and computational methods such as molecular docking, they showed that these lectins effectively blocked viral entry by interacting with mannose-rich glycans present on the SARS-CoV-2 protein. The interaction between negatively charged heparin sulphate proteoglycans and positively charged portions of the viral envelope glycoproteins is the most plausible mechanism for virus–cell attachment that has been postulated [7]. Recent years have seen research on a variety of plant and microbial lectins, such as actinomycin (AH), concanavalin-A (ConA), cyanovirin-N (CV-N), microvirin (MVN), griffithsin (GRFT), and banana lectin (BanLec). These lectins, for example, BanLec, typically have several sugar-binding sites, which enable them to interact with gp120 (glycoprotein bound) in multivalent ways [4,7]. Species from the *Allium genus*, including *Allium sativum* (garlic) and *Allium ursinum* (wild garlic), are well known for their medicinal properties, particularly their antiviral, anti-inflammatory, and antimicrobial activities. In the case of Coronaviruses, it has been shown that Urtica Dioica lectin binds N-acetylglucosamine-like residues present on glycosylated envelope glycoproteins, thus inhibiting the binding of SARS-CoV to host cells [8]. By selectively binding to the spike glycoprotein of the SARS Coronavirus (SARS-CoV), GRFT also inhibits SARS-CoV infection both in vitro and in vivo. It exhibits activity against several other coronaviruses harmful to people, animals, and birds [9].

The information spectrum method (ISM), a virtual spectroscopic method for protein analysis, is based on the fundamental electronic properties of amino acids and only requires the availability of the nucleotide sequence to study the protein. ISM is a bioinformatic approach that analyses proteins based on their amino acid sequences, converting them into a unique “spectrum” of frequencies [10]. It plays a key role in this study by enabling the analysis of protein–protein interactions between mannose-specific lectins from *Allium species* and the SARS-CoV-2 spike protein. In this study, we aimed to use a well-established method and understand how lectins can interfere with glycosylation-dependent viral mechanisms without directly analysing the carbohydrate components. The ISM provides a way to determine which regions of the spike protein are likely to interact with lectins, highlighting their potential for antiviral activity against SARS-CoV-2, focusing on protein–protein interactions. Here, we focus on the theoretical selection of mannose-specific lectins as SARS-CoV-2 entry inhibitors. Using a bioinformatic approach, we employed the Informational Spectrum Method (ISM) to identify interaction sites between these lectins and the spike protein of SARS-CoV-2, focusing on conserved glycosylation sites critical for viral entry.

## 2. Materials and Methods

### 2.1. Viruses

All mannose-specific lectin, human ACE2 (Q9BYF1), SARS-CoV (P59594) protein and HIV-1 (Q5G5V7) sequences were taken from the UniProt database (www.uniprot.org, accessed on 24 November 2024). The SARS-CoV-2 viruses are represented by the following sequences:

hCoV-19/Wuhan/Hu-1/2019, EPI_ISL_402125
hCoV-19/USA/HI-TAMC_370/2024, pango lineage KP.2.3, EPI_ISL_19322819
hCoV-19/Spain/CT-HUVH-EXE07428/2024,pangolineageKP.3.1.1, EPI_ISL_19388771

taken from the GISAID database (https://www.epicov.org/epi3/frontend#49b8c8, accessed on 24 November 2024).

### 2.2. Informational Spectrum Method (ISM)

The ISM is the virtual spectroscopy method for analysing protein–protein interactions [11,12]. This bioinformatic approach encompasses three basic steps: (i) the representation of the protein’s primary structure as a numerical sequence by assigning to each amino acid the corresponding value of the electron–ion interaction potential (EIIP), (ii) the transformation of the obtained numerical sequence into the informational spectrum (IS), and (iii) the calculation of the cross-spectrum (CS) between interacting proteins.

The EIIP is the physical parameter determining organic molecules’ long-range interactions (distances 5–1000 Å [12,13]). This molecular descriptor is defined by the following Equation (1) [14,15]:

(1) $$W=0.25\frac{Z\times \sin(1.04\pi Z^{*})}{2\pi },$$

where Z* is the average quasi-valence number (AQVN):

(2) $$Z^{*}=\frac{1}{N}\sum_{i=1}^{m}n_{i}Z_{i},$$

(2) N is the total number of atoms, n_i_ is the number of atoms of the i-th component, Z_i_ is the valence number of the atomic element in the molecule, and m is the number of components. The EIIP values calculated according to Equation (1) are given in Rybergs (Ry).

The numerical sequence, representing the primary structure of a protein, is transformed into the informational spectrum by the discrete Fourier transformation:

(3) $$X(n)=\sum_{m=1}^{N}x\left(m\right)e^{-i2\pi (m-1)/N},n=1,2,\ldots ,N/2$$

(3) X(m) represents the m-th element of a given numerical series, with N being the total number of points in that series, and X(n) is the coefficient of the discrete Fourier transformation. This transforms the information contained in the sequence of amino acids into a series of frequencies and their corresponding amplitudes. The frequencies in the informational spectrum (IS) reflect the distribution of structural motifs with specific physicochemical properties, which are crucial in defining a protein’s biological function. When comparing proteins with similar biological or biochemical functions, the informational spectrum method (ISM) can identify frequency/code pairs specific to their shared biological characteristics or related interactions. The common spectrum (CS) highlights these shared informational features of the protein sequences:

(4) $$C\left(j\right)=\prod_{i=1}^{N}S(i,j)$$

(4) C(j) refers to the j-th element of the common spectrum (CS), while S(i,j) is the j-th element of the i-th informational spectrum (IS). The standard information encoded in the primary structures of the proteins being analysed is captured by the frequencies in the CS. These frequencies correspond to the typical biological function or shared interactors of the proteins that are being examined through the ISM analysis. In the CS, the amplitude indicates the strength of the interaction, and the signal-to-noise (S/N) ratio reflects the specificity of the interaction between the two proteins.

### 2.3. Continuous Wavelet Transform (CWT)

The CWT is a mathematical tool, used to decompose a signal into wavelets, investigating the time-varying frequency spectrum. According to the definition, it computes the real continuous wavelet coefficient for each given scale presented in the scale vector and each position b from 1 to n, where n is the size of the input signal. x(t) is the input signal, and ψ is the chosen wavelet function. The continuous wavelet coefficient of x(t) at scale a and position b is as follows:

(5) $$C_{a,b}=\int x(t)\frac{1}{\sqrt{a}}\psi^{*}\left(\frac{t-b}{a}\right)dt,$$

In our case, x(t) corresponds to an EIIP value of an amino acid at the position t in the sequence, scale a corresponds to half of the amino acid sequence length, while b corresponds to the position in scale a. The output is a matrix comprising m columns and n rows, where m is the size of the scale vector a. The scale is eventually normalised to the ISM frequency range of 0.0 to 0.5. ψ is the chosen wavelet function. In this work, we used the Mexican Hat wavelet, as in the reference [16]:

(6) $$\psi (x)=\left(\frac{2}{\sqrt{3}}\right)\pi^{-\frac{1}{4}}\left(1-x^{2}\right)e^{-x^{2}}.$$

### 2.4. Sequence Alignment and Similarity

Sequences of mannose-specific proteins were aligned using Clustal Omega [17]. The corresponding alignment file was downloaded and imported into BioEdit 7.7 software [18], where the sequence similarity matrix was calculated.

## 3. Results and Discussion

Garlic, which belongs to the *Allium* species, is well known for its medical properties, such as anti-inflammatory, antibacterial, antimicrobial, and antioxidant properties [19,20,21,22]. In particular, with this study, we tried to extend its relevance to understand its antiviral properties better. The findings indicate that more and more research uses herbs popular in traditional medicine that have antiviral effects on several viral infections. From a bioinformatic perspective, this study highlights that two lectins from a widely used genus like *Allium* (garlic and wild garlic) are of particular interest, as these species are readily available in nature, safe to work with, and well studied for their medicinal properties. Several studies have addressed the potential of plant lectins to inhibit various viruses, including coronaviruses [5,23]. In this study, we used two lectins from *Allium sativum* (P83886) and *Allium ursinum* (O24427) and applied the information spectrum (ISM) method to investigate protein–protein interactions between the SARS-CoV-2 spike protein and mannose-specific lectins.

### 3.1. Identification of Mannose-Specific Lectins Interacting with SARS-CoV-2 S1 Protein Using Consensus Spectrum Analysis

**Figure 1:**
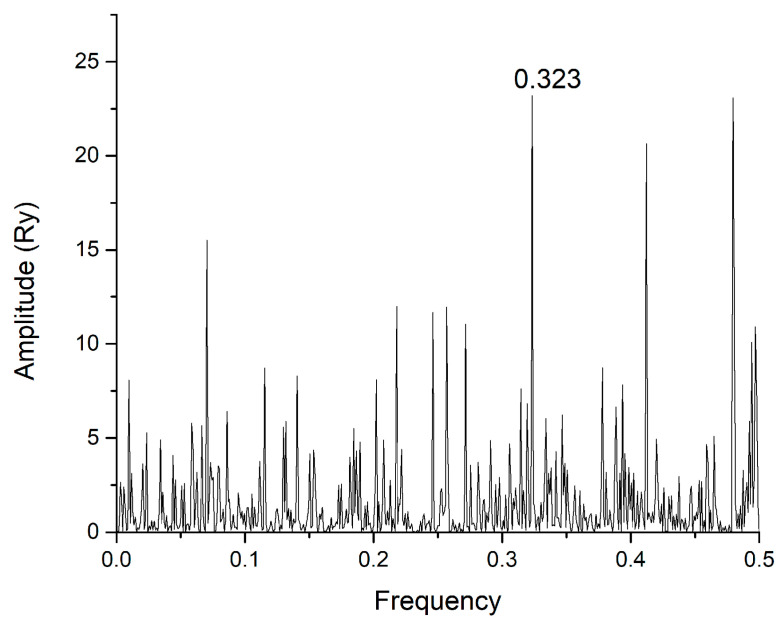
CS of SARS-CoV-2 S1 protein wild type and human ACE2 receptor.

In this study, we use the ISM-based method because of its uniqueness, as it allows the comparison of primary amino acid sequences through frequency spectra, identifying typical interaction features not apparent in traditional structural or sequence alignment methods. Using the ISM, we identified a fundamental interaction frequency, F(0.323), shared by the SARS-CoV-2 S1 protein and mannose-specific lectins (Figure 1).

**Figure 2:**
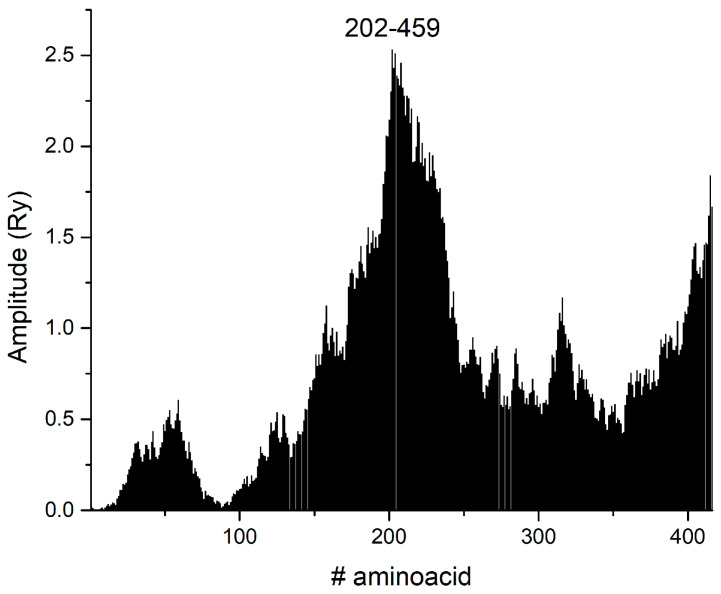
Corresponding interacting domains in SARS-CoV-2 S1 WT at F(0.323).

This type of frequency indicates that functional motifs that may mediate protein–protein and protein–carbohydrate interactions are conserved. We identified the region of interaction between lectin and the SARS-CoV-2 S1 spike protein, specifically between residues 202 and 459 (Figure 2).

The contribution of this result is enhanced by the fact that the domain 202–459 overlaps with the receptor-binding domain (RBD) (336–516) [24], which represents a critical region of the spike protein that directly binds to ACE2 (angiotensin-converting enzyme 2). Lectin binding to this domain can block glycosylation sites and interfere with the spike protein’s ability to bind to ACE2, thereby interfering with the virus entry mechanism. This region includes the RBD and glycosylated asparagine residues (N234, N282, N331, N343). The discovery of interaction frequencies specific to glycosylated regions (especially N234, the oligomannose type) advances the understanding of how protein–glycan interactions occur, where they sterically hinder binding to the oligomannose-type glycan at the N234 residue. These lectins can prevent the interaction between the spike protein and the ACE2 receptor. This is particularly important for SARS-CoV-2 viruses, where glycan shields are a known mechanism to evade host immunity, as already described by Grant et al. [24]. Our in silico study identifies essential glycosylation sites within this critical interaction domain—N234, N282, N331, and N343—focusing on N234 as an oligomannose-type glycan. This glycan has a high affinity for mannose-specific lectins, potentially making it an ideal target for therapeutic interventions. The presence of oligomannose and complex-type glycans in this region provides additional specificity to potential therapeutic molecules to block viral entry by preventing glycan-mediated interactions with host cells, which is consistent with the published data of Watanabe et al. [25]. Also, the interaction site is crucial, as it is positioned on the surface of the viral spike protein [26], making it accessible for potential therapeutic lectin targeting.

Published research on SARS-CoV-2 showed that the spike protein undergoes extensive glycosylation, with both complex and oligomannose-type glycans playing a pivotal role in evading the immune system and entering the host cell [27]. Specifically, an oligomannose-type glycan at N234 has been implicated in stabilising the spike protein and modulating its interaction with the ACE2 receptor [28]. Our manuscript is in accordance with these findings, where lectins targeting this oligomannose site can potentially inhibit virus entry—implying that oligomannose-type glycans on the spike protein are well-known targets for lectin-based inhibition strategies. Also, it was explained that mannose-binding lectins (MBLs), which are part of the innate immune response, have shown the ability to neutralise SARS-CoV-2 by binding to the glycan shield of the spike protein, preventing it from engaging with the ACE2 receptor [29]. According to these findings, we emphasise that this strengthens the case for developing lectins of plant or natural origin as therapeutic agents against SARS-CoV-2. This study highlights the lectin’s specificity for oligomannose-type glycans, which are less abundant in human host glycoproteins but more abundant in viral glycoproteins such as those on SARS-CoV-2. However, the specificity is also crucial because it minimises the risk of side effects or unwanted interactions with human glycoproteins, improving the therapeutic safety profile of these lectins.

Figure 1 presents the consensus spectrum (CS) of SARS-CoV-2 and ACE2 proteins. The dominant peak in this CS corresponds to the frequency F(0.323). To identify the possible interactors of SARS-CoV-2 S1 protein, the mannose-specific lectins from the UniProt database (https://www.uniprot.org, accessed on 24 November 2024) were screened using the ISM with the dominant peak on the frequency F0.323). The list of corresponding lectins that have a dominant peak in CS at the frequency F(0.323) is given in Table S1.

### 3.2. Structural Model of SARS-CoV-2 Spike Protein Trimer

**Figure 3:**
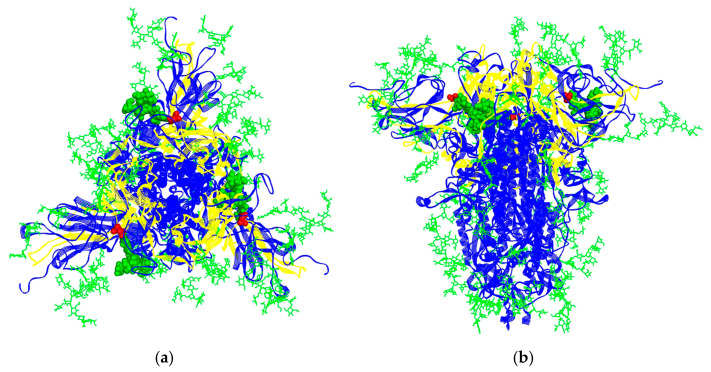
(**a**) Top and (**b**) side view of SARS-CoV-2 S1 (blue line ribbon) protein trimer with marked interaction domains determined using ISM (yellow line ribbon), N234 residues (red CPK), and glycans (green lines—all, CPK—bound to N234).

Figure 3 shows the structural model of the SARS-CoV-2 S1 fully glycosylated protein trimer, obtained from the CHARMM-GUI Archive—COVID-19 Proteins Library [30,31,32], head-only models (residue 1–1146) code 6VSB. The S1 subunit of the spike (S) protein is critical for the ability of the virus to bind to the ACE2 receptor on host cells, with distinct features such as labelled interaction domains, glycosylation sites, and glycans [33]. The RBD protein is essential for the viral entry mechanism of SARS-CoV-2 [34]. Figure 3 shows that the yellow and blue regions represent different domains or structural elements of the S1 subunit. Yellow colours represent receptor domains that are important in the molecular recognition of ACE2, while blue represents the rest of the S protein. Each chain consists of S1 and S2 subunits. The S1 subunit is responsible for receptor binding, while the S2 subunit facilitates membrane fusion. The RBD undergoes conformational changes, switching between the “up” (open) and “down” (closed) positions to enable binding to the ACE2 receptor. This figure highlights the interaction domains, presumably focused on residues critical for ACE2 binding. Glycosylation is a post-translational modification where sugar chains (glycans) are attached to specific amino acids in a protein [35]. Glycosylation also ensures the structural stability of the spike protein [28,35]. N-linked glycosylation sites (such as N234, N282, N331 and N343 in the SARS-CoV-2 spike protein) are crucial for maintaining the structural integrity of the spike protein and enhancing its interaction with host cells, which were also noticed in the manuscript published by Aloor et al. [36]. The N234 residue, marked with red spheres in Figure 3, is an asparagine residue that plays a vital role in N-linked glycosylation. This particular residue is glycosylated with oligomannose-type glycans. The image shows extensive glycan coverage (green), particularly around the spike trimers, indicating how the virus can evade detection by the host’s immune system. This also confirms a theory that, for SARS-CoV-2, glycans form a protective “shield” over the spike protein, camouflaging it from immune recognition by host antibodies. These glycans can also act as “decoys”, preventing the immune system from effectively neutralising the virus, as Watanabe et al. explained [37]. Proper glycan binding helps the protein to fold and function properly, allowing the virus to maintain its infectivity and ensure a stable *Pseudolicoriella hygida* lectin interaction with the S1 protein.

### 3.3. Mannose-Specific Lectins Candidates

**Figure 4:**
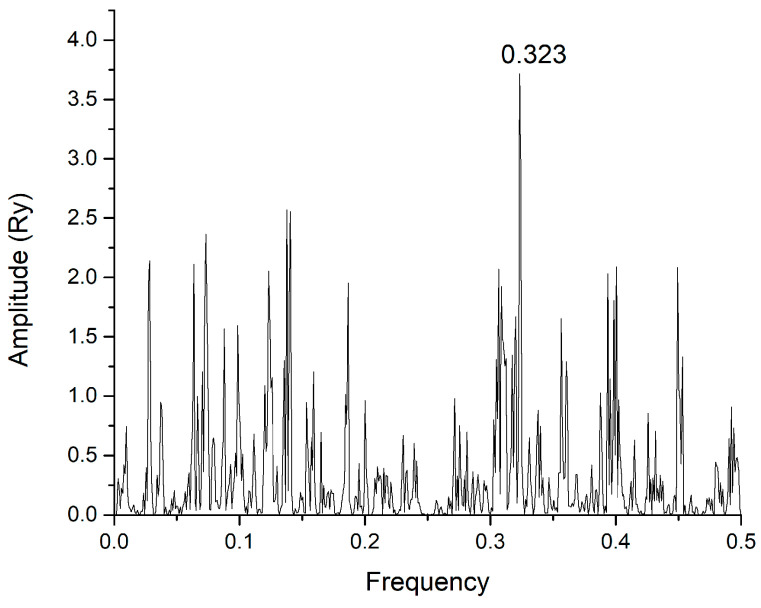
CS of SARS-CoV-2 S1 protein wild type and mannose-specific lectin from *Allium ursinum*.

**Figure 5:**
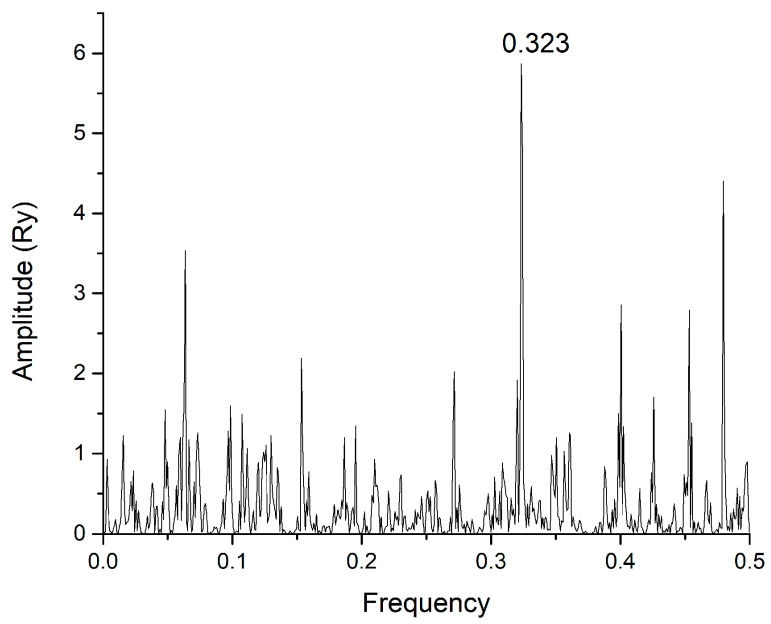
CS of SARS-CoV-2 S1 protein wild type and mannose-specific lectin from *Allium sativum*.

Regarding the lectins from the *Allium genus*, two interactors were identified: P83886 from *Allium sativum* and O24427 from *Allium ursinum*. P83886 is among the top 10% of the candidates, while O24427 is in the first third. The CS of S1WT with O24427 and P83886 is given in Figure 4 and Figure 5, respectively. The corresponding interaction domain from S1 at the frequency F(0.323) corresponds to the range 202–459 (Figure 2). It is a broad domain on the surface of the S1 protein, corresponding to the RBD (336–516) [38]. According to the site-specific glycan analysis [25], it contains the following glycosylated asparagine residues: N234, N282, N331 and N343. However, only N234 is oligomannose-type, while the rest of the asparagine residues are complex-type N-glycosylated glycans. The corresponding interaction domain with marked N234 is presented in Figure 3. This suggests that those lectins could be good SARS-CoV-2 entry inhibitor candidates.

Table S1 shows organisms from different plants, fungi, and animals with obtained amplitudes and signal-to-noise ratios (S/Ns). It is important to emphasise those lectins from the same genus (e.g., Fusarium) have relatively similar amplitude and S/N values, suggesting potentially similar taxonomic patterns. Amplitude values vary significantly among different organisms, ranging from 5.98 to 42.54, where the largest amplitude is 42.54 from *Tetrabaena socialis* (green algae), and the smallest is 5.98 from *Pseudolicoriella hygida* (flies). The data show that a higher amplitude corresponds to a higher S/N ratio, indicating a more accurate signal. However, this relationship is not linear, as some data show outliers, such as mannose-specific lectins from *Phoenix dactylifera* (date palm), with a relatively low S/N (52.83) despite moderate amplitude (19.49), while green algae have the largest amplitude (42.54) but only a moderate S/N (61.30). This may indicate that, although the signal strength is high, the background noise is also relatively high, suggesting that the interaction is less specific. The resulting mannose-specific lectins recognise and interact with high-mannose glycan structures commonly found on the surface of plant cells and in glycoproteins, which is already partially explained in the manuscript by Barre et al. [39] on *Allium sativum* but not on *Allium ursinum*.

The difference in amplitude between *Allium sativum* (30.123) and *Allium ursinum* (18.45467) suggests that garlic lectins may have a stronger binding affinity or higher glycan binding capacity than wild garlic lectins. In mannose-specific lectin interactions, the difference in S/N ratios (74.91202 vs. 46.5886) suggests that garlic lectin shows higher specificity towards mannose-rich glycans. *Allium sativum* shows a more robust and distinct binding of mannose-specific lectins, which may reflect a more significant amount or better availability of mannose-rich glycans. This also means that garlic’s lectin and glycan-binding signal is more accurate, with less background noise, making the interaction more detectable and precise. The lower S/N for *Allium ursinum* indicates that, although it also has mannose-rich glycans, mannose-specific lectins may be less abundant or less efficiently recognised. This effect may be due to differences in glycan complexity, structural variations, or lower glycoprotein expression. This could reflect subtle differences in the plant’s biological role or how the glycoproteins interact with external factors, such as viruses or immune cells. This could lead to a more effective potential of *Allium sativum* lectin in interfering with SARS-CoV-2 glycosylation sites. Mannose-specific lectins from *Allium sativum* and *Allium ursinum* could theoretically block the interaction between the spike protein and the ACE2 receptor by binding to high-mannose glycan sites on the spike protein. *Allium sativum* lectins, given their higher amplitude and more accurate S/N ratio, may have a more significant inhibitory effect on the SARS-CoV-2 spike protein due to their greater capacity to bind mannose glycans.

**Figure 6:**
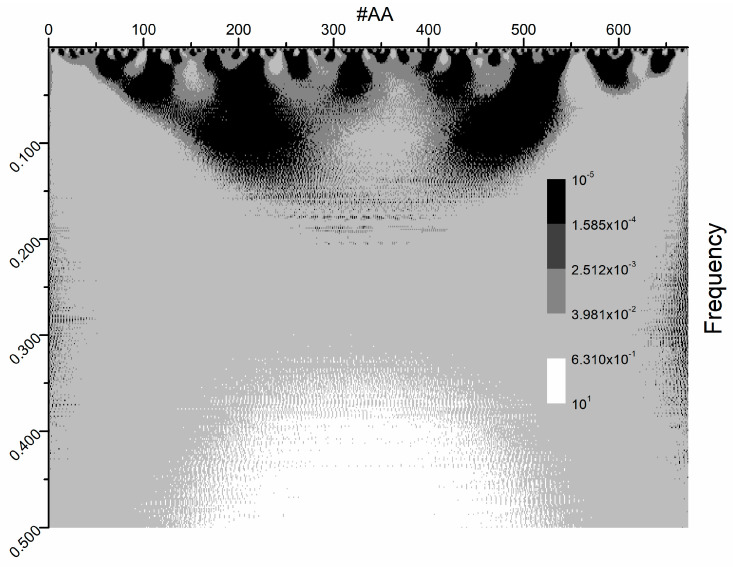
CWT plot of SARS-CoV-2 S1 protein wild type at all ISM frequencies.

**Figure 7:**
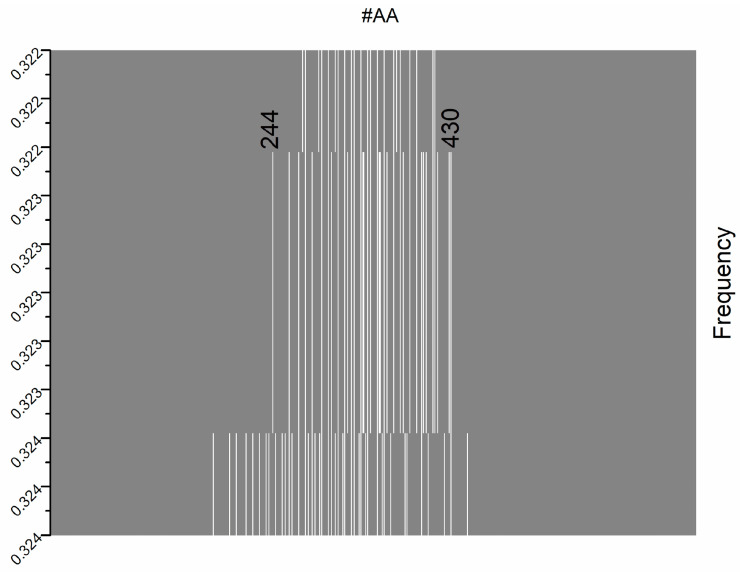
CWT spectrum of SARS-CoV-2 S1 protein wild type at ISM frequency F(0.323). Grey: wavelet coefficients ≤ cutoff 0.5975; white: wavelet coefficients > cutoff 0.5975.

The CWT analysis of the S1 protein sequence is presented in Figure 6 and Figure 7. To present the results more clearly, instead of direct wavelet coefficient values, their log_10_ values are used. In addition, the wavelet heatmap is presented in five shades (Figure 6). However, to find the proper range from the wavelet transform at F(0.323), the obtained wavelet coefficient curve had to be fitted with a polynomial function. The residual values of wavelet coefficients are calculated as the difference between the wavelet coefficients and the fitted polynomial function (Figure S1A). The coefficient cutoff is defined as the sum of the positive residuals’ average value and the fitted function’s maximum value (Figure S1B). The range is determined by the positions for which the coefficients are above the specified cutoff.

The isolated plot at ISM frequency F(0.323) is presented in Figure 7. Notably, the “hotspot” amino acid residues are in the range of 244–430 (Figure S1C).

Compared to the ISM domain (Figure 2), the size of the interaction domain is narrower, and one can determine the discrete values of the amino acid positions. However, the advantage of the ISM approach over the wavelet is in the mapping of the secondary interaction domains. In Figure 2, there are several peaks with lower amplitudes versus the CWT approach, where only a global maximum is present with several discrete values.

The results shown in Figure 6 and Figure 7 highlight the specificity of the interactions between the tested S1 subunit and any lectin at F(0.323) (Table S2). A trend is also observed in Figure S1B, supporting this conclusion, where the peak represents a specific high-affinity binding region of the S1 subunit to which the lectins bind.

In Figure 7, the frequency distribution further supports the specific and uniform binding behaviour of lectins. The data are tightly clustered within a narrow range (0.323–0.324), reflecting recent and precise interactions. The central region (244–430) closely corresponds to the specific part of the glycan target where the lectin makes maximum binding, highlighting the structural precision in its interaction with the viral spike or host receptor.

According to studies [40,41] that used hydrophobic templates, which successfully demonstrated the generation of ligands to modulate proteins such as β-galactosidase, and considering our SARS-CoV-2 study of glycosylation-dependent protein–protein interactions involving the hydrophobic regions of proteins, we highlight the potential of designing vector templates for glycan-binding sites as a next step toward discovering novel lectin candidates that could selectively interfere with or monitor viral binding and entry.

### 3.4. Sequence Similarity of Mannose-Specific Lectins

The sequence alignment of the S1 protein and mannose-specific binding lectins is presented in Figure S2. A motif from both lectins showing the highest similarity to the S1 glycoprotein is GNGNY, corresponding to GNYNY, domain 434–438, part of the RBD. This motif is also conserved in the spike glycoprotein of SARS-CoV (P59594). Regarding the SARS-CoV-2 strains KP.2.3 and KP.3.1.1, the corresponding motif is GNYDY, with the N to D mutation considered conservative.

The sequence similarity of all mannose-specific lectin candidates is provided in Supplementary Table S2. Among the Allium lectins, the highest similarity is observed between P83886 (*Allium sativum*) and A0A0S2VEQ1 (*Curcuma longa*), with a score of 0.977. Another lectin, O24427 from *Allium ursinum*, shares a similarity score of 0.607 with both P83886 and A0A0S2VEQ1. Two distinct clusters of lectins were identified based on the amplitude, signal-to-noise ratio (S/N), and similarity scores. The first cluster comprises top candidates with amplitude values ranging from 28.67 to 30.12, S/N values between 59.84 and 74.91, and a minimum similarity score 0.95. This cluster includes lectins P83886 (*Allium sativum*), A0A0S2VEK0 (*Curcuma amada*), A0A0S2VEQ1 (*Curcuma longa*), and A0A0S2VEJ7 (*Zingiber officinale*). Despite originating from different organisms, these lectins demonstrate high affinity and selectivity toward the S1 glycoprotein in silico. The second cluster is characterised by amplitude values of 23.56 to 25.45, S/N values between 49.41 and 52.94, and a minimum similarity score of 0.984. This cluster exclusively consists of lectins from *Mycobacteroides abscessus*, including A0A1T7XQE7, R4ULX2, A0A1U1ABQ1, A0A8B4DUW7, A0A9Q7SDL5, A0A0U0ZSS4, A0A9Q7SNH7, and B1MB33. The high similarity scores within this cluster reflect their shared taxonomic origin, as shown in Table S1.

### 3.5. Justification of the ISM Approach

Klevansky et al. investigated the antiviral potential of the lectin concanavalin A (ConA) against SARS-CoV-2 [42]. This study uses super-resolution dSTORM microscopy to visualise the interaction of ConA with virions, demonstrating that concanavalin possesses antiviral and virucidal mechanisms of action. The manuscript emphasises the need for high S/N ratios in imaging techniques such as dSTORM to reliably visualise the interaction between lectins and viral particles. The authors confirmed their hypothesis on several levels, with an IC50 of 1.39 μg/mL for ConA and a selectivity index (SI) exceeding 299, indicating potent antiviral activity and low cytotoxicity. These values highlight ConA’s strong affinity and binding efficiency and clearly demonstrate that ConA blocks an early step in the infection cycle by emphasising its strong binding to the S1 domain of the spike protein (and even to Alpha, Beta, Delta, and Omicron variants).

**Figure 8:**
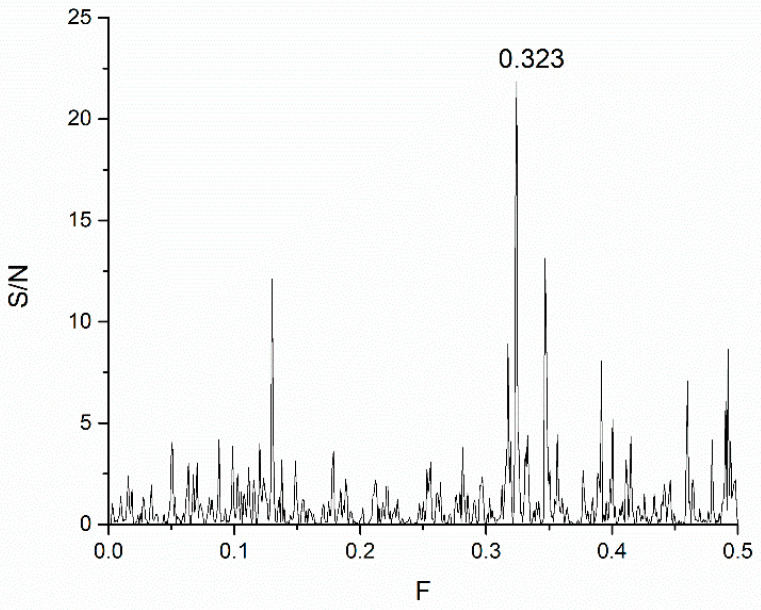
CS of SARS-CoV-2 wild type S1 protein and lectin Concanavalin A P81461.

In Figure 8, the CS of the SARS-CoV-2 wild type S1 and Concanavalin A P81461 is presented. We found, by our method (Table S3), searching the whole UNIPROT database, four Concanavalins A: P81461, P81460, P55915, and P14894. This definitely serves as proof of concept in our study of S1 protein–lectin interactions at ISM frequency F(0.323).

**Figure 9:**
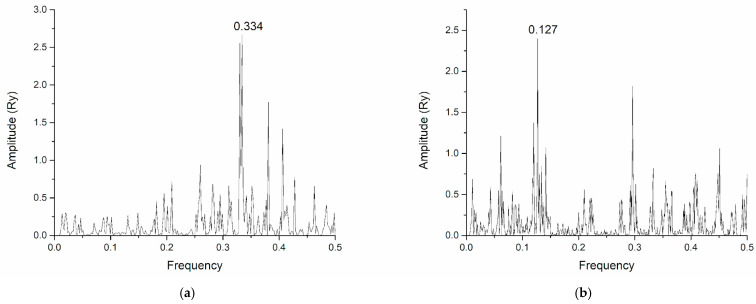
(**a**) CS of HIV-1 gp120 envelope protein and lectin GRFT. (**b**) CS of H1N1 Hemagglutinin and GNL.

**Figure 10:**
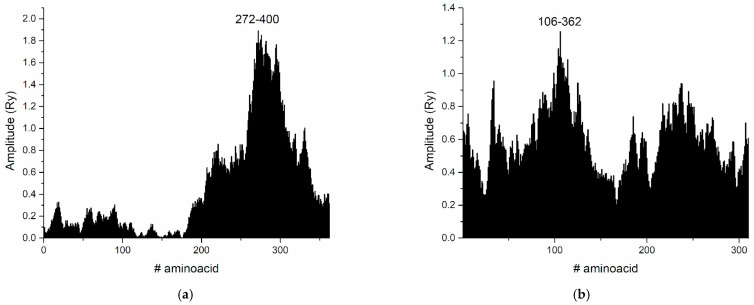
(**a**) Corresponding interacting domains in HIV-1 gp120 at F(0.334). (**b**) Corresponding interacting domain in Hemagglutinin H1N1 at F(0.127).

As we pointed out in the Introduction, studies on the interactions between lectins and other viral proteins have been reported. In a work conducted by Alexandre et al., the lectin GRFT (griffithsin) was experimentally observed to bind to gp120 [43]. The glycan at position N386 was identified as playing a key role in gp120-lectin binding. Figure 9 presents the CS spectra of the HIV-1 gp120 envelope protein and the lectin GRFT, and H1N1 Hemagglutinin and GNL (*Galanthus nivalis* lectin), respectively. Figure 10 presents the corresponding interacting regions in gp120 and Hemagglutinin, respectively.

The common frequency is at F(0.334), and the corresponding interaction domain in gp120 is 272–400. According to the references, olligomanose-rich glycosylated asparagine residues in gp120 are the following: N156, N234, N262, N295, N332, N339, N363, N386, N392 and N448. Therefore, it is clear that the key domain for intermolecular recognition spans the olligomanose sites, including the key glycan position N386, proving the interaction with the lectin molecule. In the case of Hemagglutinin H1N1, high mannose positions are N21, N32, N91, N378, N289, N483. This supports our application of ISM to the glycosylated proteins and highlights how glycosylation modulates the binding affinity and specificity between gp120 and GRFT, contributing to the antiviral potential of the lectin against HIV-1. High-mannose glycosylation sites in hemagglutinin have been identified at N21, N32, N91, N378, N289, and N483. These positions play a role in GNL binding, indicating that glycans attached to these asparagine residues are likely important for the ability of GNL to bind hemagglutinin and potentially inhibit virus activity. The ISM approach, by identifying specific interaction frequencies, provides insight into which protein regions are likely to mediate critical protein–protein interactions. In this study, ISM pinpointed the interaction regions in gp120 and hemagglutinin, highlighting its utility as a bioinformatic tool for mapping protein–protein interfaces. The ISM analysis of HIV-1 gp120 and GRFT identified a key frequency at F(0.334) corresponding to the interaction domain between residues 272–400. This domain includes conserved motifs that are key to the ability of the gp120 protein to interact with other proteins or ligands, such as GRFT. The interaction domain includes structural motifs that facilitate GRFT binding to gp120, which may interfere with the role of gp120 in binding to host cell receptors such as CD4 and co-receptors. By binding to these motifs, GRFT can potentially block the interaction between HIV-1 gp120 and the host cell receptor, thereby inhibiting viral entry.

## 4. Conclusions

In this manuscript, we have presented a bioinformatic analysis that demonstrates the potential of mannose-specific lectins from the *Allium genus*, particularly *Allium sativum* and *Allium ursinum*, as potential inhibitors of SARS-CoV-2, as mannose binds with high affinity to ACE2 receptors, which are responsible for the entry of the virus into the cell. Using the information spectrum method (ISM), we identified vital interaction frequencies between the spike protein of SARS-CoV-2 and these lectins, especially in regions crucial for virus entry, such as the receptor-binding domain (RBD). This result indicates possible interactions between these proteins and the S1 glycoprotein, especially in the oligomannose residues of N-glycosylated asparagine (N-234). Lectin candidates from the genus *Allium* are in the top-third of proposed lectins, suggesting potential inhibitors of SARS-CoV-2 entry. We also emphasise that further experimental validation is necessary to confirm these in silico results and explore the possible therapeutic applications of these lectins.

It is a long-range molecular interaction method that takes into account only protein sequences and determines domains responsible for intermolecular recognition. From these results, it is clear that amino acid glycosylation does not affect the electronic parameters of the amino acid residues, but the glycosylation itself of certain protein domains is instead an additional modulation of protein–protein interactions.

## Data Availability

The original contributions presented in this study are included in this article and Supplementary Materials; further inquiries can be directed to the corresponding authors.

## Supplementary Materials

The following supporting information can be downloaded at https://www.mdpi.com/article/10.3390/life15020162/s1, Figure S1: (A) Residuals from polynomial fit of wavelet coefficients at F(0.323) in CWT of SARS-CoV-2 wild type S1 sequence, (B) Polynomial fit of wavelet coefficients at F(0.323) and determination of interaction domain amino acid residues, (C) S1 domain 244–430 with red-marked amino acid residues above the cutoff; Figure S2: Sequence alignment of SARS-CoV-2 S1 glycoprotein and mannose-specific lectins; Table S1: List of mannose-specific proteins with dominant amplitude on the frequency F(0.323); Table S2: List of all proteins from the UNIPROT database with dominant amplitude on the frequency F(0.323); and Table S3: Sequence similarity of mannose-specific proteins with dominant amplitude on the frequency F(0.323), with corresponding A and S/N values.
