# Supplementary material for: Bioinformatic Selection of Mannose-Specific Lectins from Allium genus as SARS-CoV-2 Inhibitors Analysing Protein–Protein Interaction
Source: Life (Basel). 2025 Jan 23;15(2):162. doi: 10.3390/life15020162 (PMC11856470; doi:10.3390/life15020162)
Supplement: Supplementary file 1 [file life-15-00162-s001.zip › Table S1.pdf]

Table S1. List of mannose-specific proteins with dominant amplitude on the frequency (0.323)

Mannose-specific lectin (UniProt ID)	Mass (Da)	Length	Organism	Amplitude	S/N
A0A0S2VEK0	16339.5	183	*Curcuma amada*	36.36092	74.91202
A0A667XT83	11761.92	106	*Myripristis murdjan*	20.87202	74.30814
A0A0S2VEQ1	16186.19	181	*Curcuma longa*	31.19915	66.78276
A0A175WDI4	12644.94	118	*Madurella mycetomatis*	22.24325	65.90633
A0A4Z2EPA1	16975.89	150	*Liparis tanakae*	28.51316	63.83051
A0A2I4B7S8	14987.94	132	*Austrofundulus limnaeus*	25.6846	63.76294
P83886	16201.25	181	*Allium sativum*	30.123	62.81757
A0A2J7ZWM3	30686.51	285	*Tetrabaena socialis*	42.53833	61.30078
A0A0S2VEJ7	16530.66	184	*Zingiber officinale*	28.67163	59.84484
A0A6P8U0F9	13082.41	116	*Gymnodraco acuticeps*	20.87498	58.53514
A0A0U0ZSS4	19633.19	188	*Mycobacteroides abscessus*	25.45572	52.93713
A0A8B8J8A8	15076.2	134	*Phoenix dactylifera*	19.49452	52.82541
A0A8H5RQA4	12992.46	118	*Fusarium sp.* NRRL 52700	18.04501	52.31895
A0A9P5E056	13245.72	118	*Fusarium beomiforme*	16.70967	52.11433
A0A8H4K5T5	13011.46	118	*Fusarium acutatum*	17.84972	51.79209

A0A8H5NJW8	13011.46	118	*Fusarium napiforme*	17.84972	51.79209
A0A8H5NSQ0	13011.46	118	*Gibberella subglutinans*	17.84972	51.79209
A0A8H5RXQ6	13011.46	118	*Fusarium tjaetaba*	17.84972	51.79209
A0A8H5W9E3	13011.46	118	*Fusarium sp. NRRL 25303*	17.84972	51.79209
A0A8H6DH90	13011.46	118	*Fusarium mundagurra*	17.84972	51.79209
A0A1A8V052	13334.96	116	*Nothobranchius furzeri*	16.97541	51.62083
A0A3Q2VPP1	13486.27	117	*Haplochromis burtoni*	16.63397	50.96214
A0A8C6LW57	13320.94	116	*Nothobranchius furzeri*	16.61116	50.80931
A0A8H5YTH5	13083.53	118	*Fusarium globosum*	17.94852	50.68741
A0A9Q7SNH7	19647.22	188	*Mycobacteroides abscessus subsp. abscessus*	24.22303	50.6461
B1MB33	19647.22	188	*Mycobacteroides abscessus* (strain ATCC 19977 / DSM 44196 / CCUG 20993 / CI	24.22303	50.6461
A0A1U1ABQ1	19633.19	188	*Mycobacteroides abscessus subsp. massiliense*	23.82141	49.97449

A0A8B4DUW7	19633.19	188	*Mycobacteroides abscessus subsp. abscessus*	23.82141	49.97449
A0A9Q7SDL5	19633.19	188	*Mycobacteroides abscessus subsp. bolletii*	23.82141	49.97449
A0A1T7XQE7	19647.22	188	*Mycobacteroides abscessus subsp. massiliense*	23.56351	49.40987
R4ULX2	19647.22	188	*Mycobacteroides abscessus subsp. bolletii 50594*	23.56351	49.40987
A0A8J4XCK0	11872.22	105	*Clarias magur*	14.92226	49.34216
A0A917QGK4	19950.93	188	*Nocardia camponoti*	26.87789	47.90655
A0A395STG1	12976.37	119	*Fusarium sporotrichioides*	16.62446	47.63578
A0AAD9BM97	12514.91	111	*Dissostichus eleginoides*	15.99152	47.52286
O24427	17676.97	166	*Allium ursinum*	18.45467	46.5886
A0A8B9R3N9	14010.58	118	*Astyanax mexicanus*	15.32628	44.85379
W5K596	14010.58	118	*Astyanax mexicanus*	15.32628	44.85379
A0A0S2VEL8	16119.15	180	*Elettaria cardamomum*	18.77273	44.67326
A0A8T2LSW6	13957.53	118	*Astyanax mexicanus*	15.09826	44.54293

A0AAD9BLD1	12527.99	111	*Dissostichus eleginoides*	14.89894	43.92756
A0A8H5NQ91	12985.38	118	*Fusarium pseudoanthophilum*	14.23225	43.63535
A0A8H5KB13	12999.41	118	*Fusarium phyllophilum*	14.24542	43.60982
A0A3P8ZZX7	14874.97	136	*Esox lucius*	16.57783	43.47387
A0A6Q2ZIK0	14858.91	136	*Esox lucius*	16.57783	43.47387
A0AAD9EXS1	13617.35	120	*Dissostichus eleginoides*	13.40841	43.18598
A0A6P7I2F0	12297.74	110	*Parambassis ranga*	12.05419	42.6831
A0A6Q2Z1F8	12149.83	111	*Esox lucius*	14.51488	42.03321
A0A559LHC4	12991.47	118	*Fusarium oxysporum f. sp. cubense*	14.39744	41.78028
A0A8J5Q1M8	12991.47	118	*Fusarium oxysporum f. sp. raphani*	14.39744	41.78028
N4UPZ9	12991.47	118	*Fusarium oxysporum f. sp. cubense* (strain race 1)	14.39744	41.78028
A0A8H5UBY3	12989.5	118	*Fusarium denticulatum*	14.18461	41.76883
A0A3P9NG84	13396.85	116	*Poecilia reticulata*	13.50361	41.55155
A0A8H5PVB8	12977.45	118	*Fusarium pseudocircinatum*	14.22144	41.47861

A0A8H5UGP8	12977.45	118	*Fusarium circinatum*	14.22144	41.47861
A0A9P5EFL9	12977.45	118	*Fusarium agapanthi*	14.22144	41.47861
A0A673CQC4	16401.47	168	*Sphaeramia orbicularis*	17.9256	41.24201
A0A8J5U4G0	13007.47	118	*Fusarium oxysporum f. sp. rapae*	13.69459	41.20022
A0A8H5IBH6	12963.42	118	*Fusarium mexicanum*	14.07021	41.16643
A0AA47NLL8	28106.47	243	*Merluccius polli*	35.38508	40.71521
A0A4Z2EGK3	13160.71	116	*Liparis tanakae*	12.69287	40.63141
A2SVT1	16344.01	185	*Chimonanthus praecox*	19.57545	39.96337
A0A6J2RW39	13233.79	116	*Cottoperca gobio*	13.2768	39.84361
A0A6I9RRF1	26993.59	264	*Elaeis guineensis var. tenera*	26.01789	39.38126
A0A8H4K6S9	13362.75	119	*Fusarium austroafricanum*	12.75003	37.96887
A0A8H4L396	10522.3	93	*Fusarium albosuccineum*	9.349	37.11176
A0A672IX59	13580.25	118	*Salarias fasciatus*	13.24018	35.55021
A0A2J8A1R8	18526.57	163	*Tetrabaena socialis*	14.7209	35.49216
A0A8K0IZE7	27054.3	270	*Cocos nucifera*	26.4346	35.17248
N1RZP8	12979.42	118	*Fusarium oxysporum f. sp.*	11.17801	34.25353

			cubense (strain race 4)		
A0A2I0VX94	15873.73	173	*Dendrobium catenatum*	14.8786	34.10083
A0A6J2RTB0	13175.75	116	*Cottoperca gobio*	11.11158	33.94948
A0A8C9Y5E5	13492.21	117	*Sander lucioperca*	11.00589	33.04049
A0A3B4YSH0	13376.97	116	*Seriola lalandi dorsalis*	9.70616	32.9784
A0A8N4FBF0	11062.23	132	*Elaeis guineensis var. tenera*	11.47098	32.43548
A0A0S2VFB7	16,208	152	*Alpinia galanga*	11.52377	32.36335
A0A8B7CFZ6	27534.1	267	*Phoenix dactylifera*	20.94837	31.82435
A0A8K0ITP4	27509.05	267	*Cocos nucifera*	21.74427	31.70402
A0A443PBP6	18380.78	173	*Cinnamomum micranthum f. kanehirae*	13.65423	31.62902
A0A3P9A174	15000.08	136	*Esox lucius*	11.35364	31.52481
P86184	25326.05	237	*Cymbosema roseum*	21.0902	31.44313
A0A087YMP9	13390.88	116	*Poecilia formosa*	10.36674	31.31905
A0A1S3Q820	13677.69	116	*Salmo salar*	11.28564	31.26716
A0A3B3VP11	13404.9	116	*Poecilia latipinna*	10.33798	31.13813
A0A6P4BF50	25752.68	234	*Arachis duranensis*	21.52146	31.01042
A0A8C9Y3E8	13464.2	117	*Sander lucioperca*	10.0916	30.76081
A0A2I0VLD5	14003.36	157	*Dendrobium catenatum*	12.60936	30.46273

A0A3Q3LPW3	13286.93	116	*Mastacembelus armatus*	9.65813	29.97682
A0A8K0N107	15608.42	172	*Cocos nucifera*	11.59526	29.23042
A0A8B7CFJ7	29607.32	274	*Phoenix dactylifera*	21.39062	29.19031
A0A8B7CFQ3	28485.96	265	*Phoenix dactylifera*	19.79384	29.06717
A0A1D1YX83	20188.5	224	*Anthurium amnicola*	16.21849	28.76165
A0A6J2UST7	13987.7	118	*Chanos chanos*	8.83667	28.29477
A0A9C6TJR5	21622.99	199	*Arachis duranensis*	16.0866	28.02539
A0A6I9QKD0	15402.26	171	*Elaeis guineensis var. tenera*	10.34309	27.80071
A0A2J7ZGQ8	26929.14	245	*Tetrabaena socialis*	16.64102	27.42826
A0A6N4UNE4	20847.97	197	*Mycolicibacterium alvei*	12.86077	27.24145
D5MNX4	25290.02	237	*Cymbosema roseum*	18.46781	27.0598
A0A2I0VLE0	13973.9	160	*Dendrobium catenatum*	10.73572	26.86786
A0A6P3VNG6	14005.78	118	*Clupea harengus*	8.13159	26.79114
A0A087Y1V9	13393.03	116	*Poecilia formosa*	7.49524	26.05655
A0A3B3W1L4	13393.03	116	*Poecilia latipinna*	7.49524	26.05655
A0A3B1KJ23	12798.18	112	*Astyanax mexicanus*	9.05521	24.80367
A0A8T2KSK8	12798.18	112	*Astyanax mexicanus*	9.05521	24.80367
A0A8C9Y4Y8	13484.2	117	*Sander lucioperca*	8.51878	24.32215

A0A9Y4KGH0	13086.57	117	*Stegastes partitus*	8.18573	24.09144
A0A6G6AC25	22447.13	216	*Borely moumouvirus*	11.62793	22.63301
A0A9Q0N6B2	12246.67	106	*Pseudolycoriella hygida*	5.97649	22.57241
A0A3Q2PY45	13343.8	117	*Fundulus heteroclitus*	6.16796	22.26238
A0A8C9Y3F9	13456.18	117	*Sander lucioperca*	7.42078	21.76186
A0AAD9BEC7	12855.44	115	*Dissostichus eleginoides*	6.13715	20.84588
A0A8C9Y458	15288.42	132	*Sander lucioperca*	7.41649	20.78942
A0A6I9R6F6	15420.32	171	*Elaeis guineensis var. tenera*	7.30493	20.3431
A0A2I0VA69	15981.72	177	*Dendrobium catenatum*	7.13995	19.5613
A0A2I0WZ74	16985.36	159	*Dendrobium catenatum*	7.65917	18.14444
