# Supplementary figures and images for: Bioinformatic Selection of Mannose-Specific Lectins from Allium genus as SARS-CoV-2 Inhibitors Analysing Protein–Protein Interaction
Source: Life (Basel). 2025 Jan 23;15(2):162. doi: 10.3390/life15020162 (PMC11856470; doi:10.3390/life15020162)

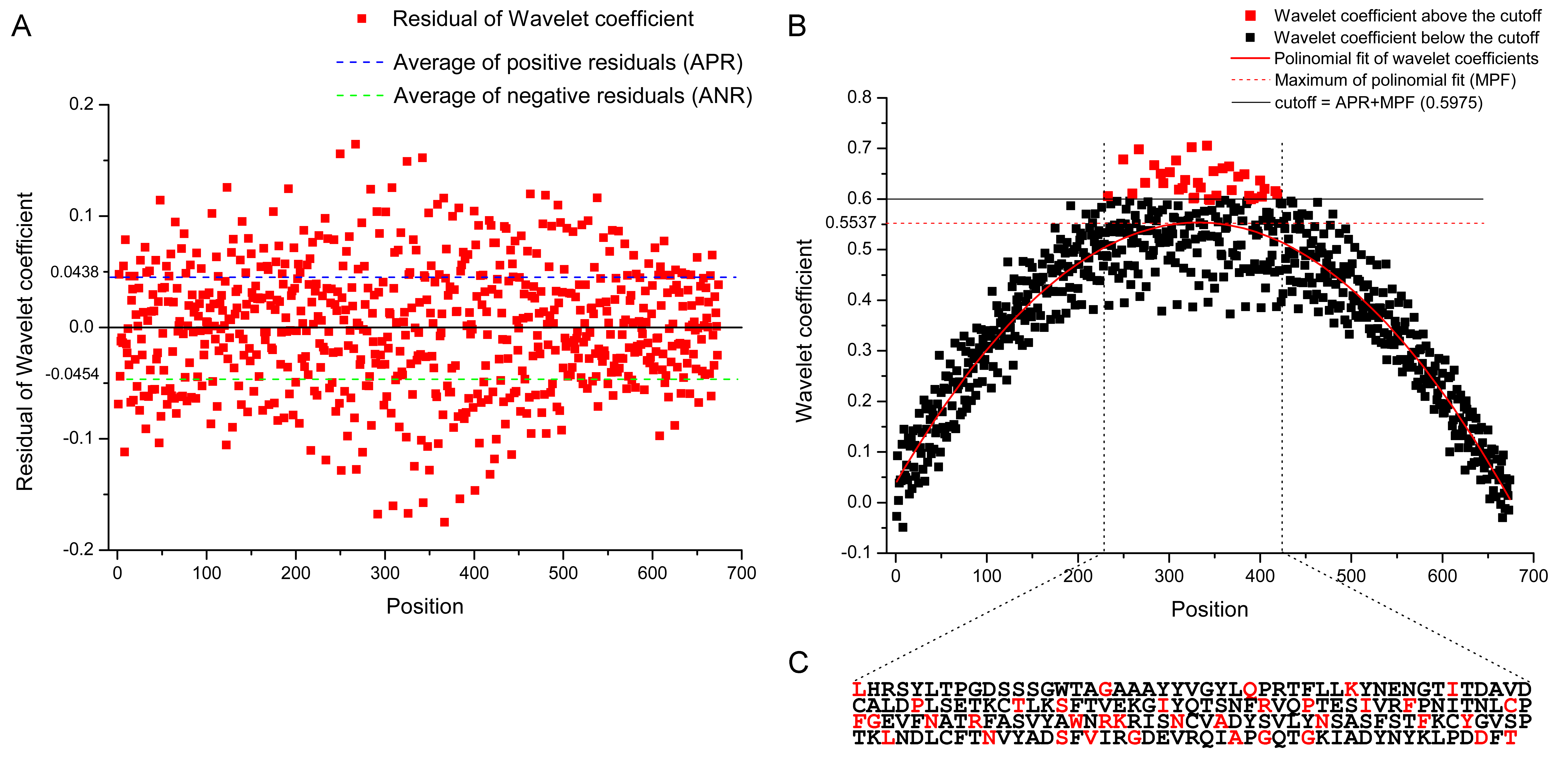

Supplement: Supplementary file 1 [file life-15-00162-s001.zip › FigureS1.tif]

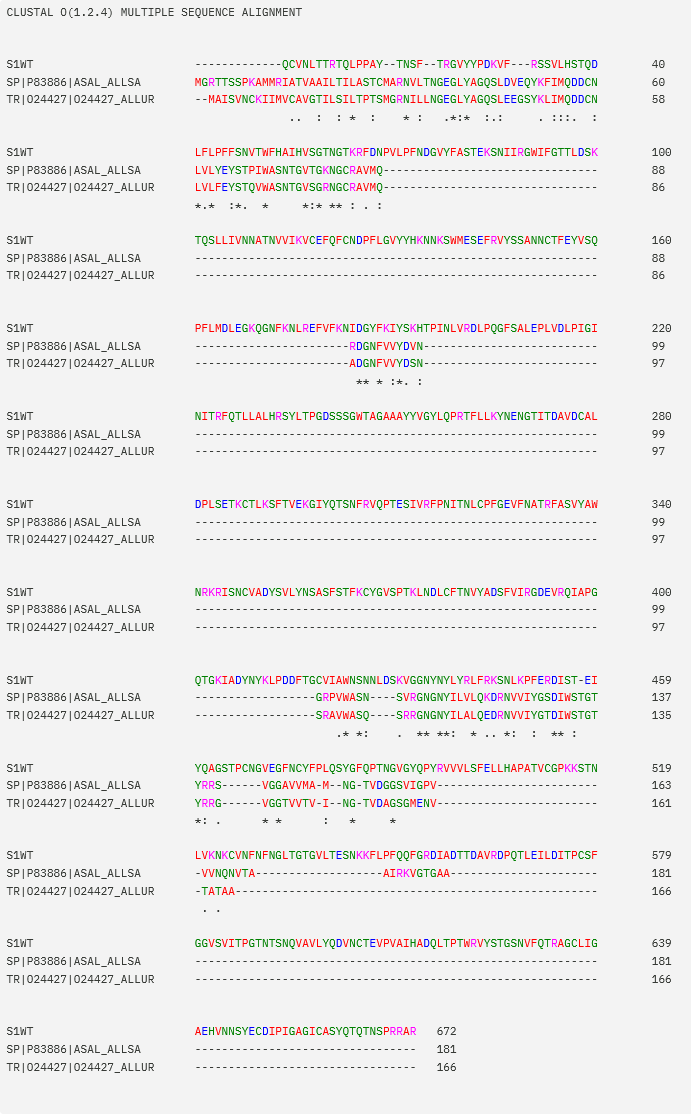

Supplement: Supplementary file 1 [file life-15-00162-s001.zip › FigureS2.png]
